# Wool Keratin-Based Nanofibres—In Vitro Validation

**DOI:** 10.3390/bioengineering8120224

**Published:** 2021-12-18

**Authors:** Diego Omar Sanchez Ramirez, Iriczalli Cruz-Maya, Claudia Vineis, Vincenzo Guarino, Cinzia Tonetti, Alessio Varesano

**Affiliations:** 1 National Research Council-Institute of Intelligent Industrial Technologies and Systems for Advanced Manufacturing (CNR-STIIMA), Corso Giuseppe Pella 16, 13900 Biella, Italy; claudia.vineis@stiima.cnr.it (C.V.); cinzia.tonetti@stiima.cnr.it (C.T.); alessio.varesano@stiima.cnr.it (A.V.); 2 National Research Council-Institute for Polymers, Composites and Biomaterials (CNR-IPCB), Mostra d’Oltremare, Pad. 20, V.le J.F. Kennedy 54, 80125 Napoli, Italy; cdiriczalli@gmail.com

**Keywords:** wool-keratin, electrospinning, cell growth, scaffolds

## Abstract

Protein-based nanofibres are commonly used in the biomedical field to support cell growth. For this study, the cell viability of wool keratin-based nanofibres was tested. Membranes were obtained by electrospinning using formic acid, hexafluoroisopropanol, and water as solvents. For aqueous solutions, polyethylene oxide blended with keratin was employed, and their use to support in vitro cell interactions was also validated. Morphological characterization and secondary structure quantification were carried out by SEM and FTIR analyses. Although formic acid produced the best nanofibres from a morphological point of view, the results showed a better response to cell proliferation after 14 days in the case of fibres from hexafluoroisopropanol solution. Polyethylene oxide in keratin nanofibres was demonstrated, over time, to influence in vitro cell interactions, modifying membranes-wettability and reducing the contact between keratin chains and water molecules, respectively.

## 1. Introduction

Keratin is a natural protein extensively used to produce scaffolds thanks to its biocompatibility and biodegradability [1]. In the past, this protein has been processed in different ways to obtain protein-based materials like films, sponges, hydrogels, and nanofibres (NFs) [2]. In particular, the ease to process keratin NFs is currently gaining relevant interest in the biomedical field, due to their ability to mimic the fibrillary structure of the extra cellular matrix (ECM). For example, keratin-NFs have reported promising results in dental implants [1,3].

Recent studies have demonstrated that the peculiar structure of proteins may influence their interaction with cells, thus addressing their fate in vitro (i.e., collagen denaturation due to partial hydrolysis). In the case of keratin, previous works have reported that extraction and manufacturing methods play a relevant role in the protein chemical structure at the sub-micro scale [4], significantly influencing the relative amino-acid contents in RGD-like sequences (i.e., Arg–Gly–Asp), thus affecting basic biological mechanisms such as cell adhesion [5].

As reported in previous studies, the use of different solvents and the integration of additional polymers in solution can affect the micro and nanostructural properties of NFs, thus altering the local interface with cells [6]. As a function of the solvent volatility, polymeric solutions may change their polymer concentration, viscosity, surface tension, and electrical permittivity during the fibre deposition, with different effects on micro- and macroscopic properties of membranes [7]. In the case of proteins like keratin, solvent interactions can modify the spatial organization of macromolecular chains, playing an essential role in the formation of supramolecular structures [8].

Besides, several studies have broadly demonstrated that some solvents can be undesirable during the production of protein-based NFs, due to the presence of low volatile residual solvent with aggressive moieties that tend to be hardly removed from fibres [9], and, in some cases, altering bioactive chemical functionalities suitable for cells recognition [10]. All previous critical aspects may damage the native bio-functionality of keratin, univocally compromising the ultimate bioactive performance of NFs.

In this context, the integration of synthetic polymers with recognized properties in the biomedical field in terms of non-toxic response (i.e., aliphatic polyesters, including polycaprolactone [11,12], polylactide acid [13], and polyalkanoates [14]) may represent a consolidated strategy to improve problems in terms of solvent stability, mechanical weakness, and structural integrity [15]. More recently, keratin has also been blended with hydrogel-like polymers, such as polyethylene oxide (PEO) [16] or polyvinyl alcohol (PVA) [17], showing good results in terms of cell interactions.

This work aims to validate the cell viability of wool keratin-based NFs for in vitro studies. Herein, it is proposed to investigate the morphological and structural properties of keratin-NFs as a function of solvent used, namely formic acid (FA), hexafluoroisopropanol (HFIP), and water. Moreover, a synthetic water-soluble polymer such as PEO will also be considered to validate its use to support in vitro cell interactions.

## 2. Materials and Methods

### 2.1. Materials

Wool fibres were used to obtain keratin powder (KS) by sulfitolysis, as reported in previous works [18]. Unless otherwise specified, PEO (average Mw 400 kDa) and all other reagents were acquired from Sigma-Aldrich (Milan, Italy).

### 2.2. NFs-Membrane Production

Polymeric solutions made of KS (in FA, HFIP and water) were employed to produce NFs-membranes by a single jet electrospinning prototype. It consists of a flat plate collector, a KDS 200 high-precision syringe pump (KD Scientific Inc., USA), a stainless steel needle, and an SL50 high-voltage generator (Spellman, UK). Table 1 reports the working conditions to produce NFs-membranes on polypropylene nonwovens. All samples were post-treated to prevent the water solubilisation of proteins by heating the samples in an oven for 2 h at 180 °C in air [16,18].

### 2.3. Morphological Characterization (SEM)

The morphology of NFs was observed using a XEVO series scanning electron microscope (Carl Zeiss Microscopy GmbH) with an acceleration voltage of 15 kV at about 30 mm working distance. The samples were sputter-coated with a 20 nm-thick gold layer in rarefied argon (20 Pa), using a Quorum SC7620 sputter coater, with a current of 20 mA for 120 s. The software Fiji-ImageJ 1.51 was used on SEM images to estimate the diameter distribution of NFs-membranes.

### 2.4. FTIR Analyses

A thermo nicolet nexus spectrometer with a smart endurance accessory was employed to record all spectra in ATR (4 cm^−1^ band resolution and 50 scans) between 4000 and 650 cm^−1^.

The peak of Amide I was fitted with Gaussians (peak analyser–OriginLab Pro 8.0) to quantify the content of secondary structures in KS. A baseline between Amide I and Amide II peaks was also used. Furthermore, the second derivate method with a quadratic Savitzky-Golay smoothing (third-order polynomial with five points) was employed to determine the wavenumbers. The frequencies were assigned as follow: 1611, 1618, 1625, and 1695 cm^−1^ to intermolecular β–sheets [19,20,21]; 1674 cm^−1^ to intramolecular β–sheets [22]; the bands between 1630 and 1640 cm^−1^ can be assigned to inter and/or intramolecular β–sheets [22,23], and were called “β–sheets II” to express the presence of bands in this region; 1668, 1682 and 1689 cm^−1^ to β–turns [20,21]; 1645, 1651, 1659 cm^−1^ to α–helix [24,25,26]; 1645, 1651 and 1659 cm^−1^ to random coil [24,25]. Moreover, the small bands of side-chains (1594 and 1604 cm^−1^) and the bands of carboxylic groups (1714, 1725 and 1731 cm^−1^) were also taken into account [24,27,28]; however, those areas were excluded in the quantification of secondary structures. As reported in the literature, the fitting was carried out limiting the values of full width at half maximum (FWHM) between 10 and 30 cm^−1^, fixing the band position and allowing any positive value for the height of Gaussians [24].

### 2.5. Cell Culture Tests

For in vitro assays, human mesenchymal stem cells (hMSCs, SCC034 from Sigma-Aldrich) were used. hMSCs were cultured in a 75 cm^2^ cell culture flask in Eagle’s alpha minimum essential medium (α–MEM) supplemented with 10% fetal bovine serum (Sigma-Aldrich), antibiotic solution (streptomycin 100 µg mL^−1^ and penicillin 100 U mL^−1^, Sigma-Aldrich) and 2 mM of L-glutamine, incubated at 37 °C in a humidified atmosphere with 5% CO_2_ and 95% air. hMSCs from 4–6 passages were used for cell proliferation assays. Before in vitro studies, membranes were cut to put in 96-well cell culture, washed and sterilized in 70% ethanol for 30 min before being washed three times with phosphate-buffered saline (PBS). hMSCs were seeded at 6 × 10^3^ onto KSFA, KSPEO and KSHFIP fibres for proliferation assays assessed via cell counting kit-8 reagent (CCK-8; Dojindo Laboratories) up to 14 days. Briefly, cell culture media was removed and changed at each time point by 100 µL of fresh medium with 10 µL of CCK-8 reagent per well and incubated for 4 h in standard conditions. The supernatant was collected, and absorbance was measured at 450 nm using a microplate reader. Results are presented as mean ± standard deviation (n = 3). Analysis of variance (ANOVA) with Tukey’s post hoc test was used to detect differences between groups. A value of *p* < 0.05 was considered to determine statistically significant differences.

## 3. Results

**Figure 1:**
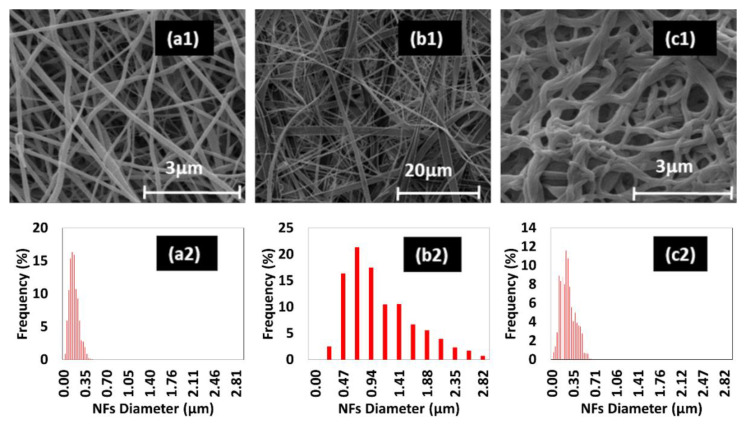
SEM images (number 1) and diameter distribution of NFs (number 2) for samples after post-treatment: (**a**) KSFA, (**b**) KSHFIP, (**c**) KSPEO-180 °C and 24 h in water.

Over the last few years, the use of natural proteins has been variously applied to the fabrication of fibrous scaffolds with improved surface properties in terms of bio-recognition and adhesiveness that identify key features for the fabrication of platforms suitable for in vitro cell interaction studies [1,29]. In particular, KS from wool has proved to be an interesting biomaterial for the fabrication of electrospun scaffolds in tissue engineering [12,29]. In this work, the morphological and structural properties of KS-NFs as a function of solvent used, namely FA, HFIP and water, were investigated. In the water case, PEO—a synthetic water-soluble polymer—was combined with KS to produce KSPEO-NFs from an aqueous solution. Their use to support in vitro cell interactions was validated. The morphology of all samples was investigated by SEM and image analysis, as shown in Figure 1.

**Figure 2:**
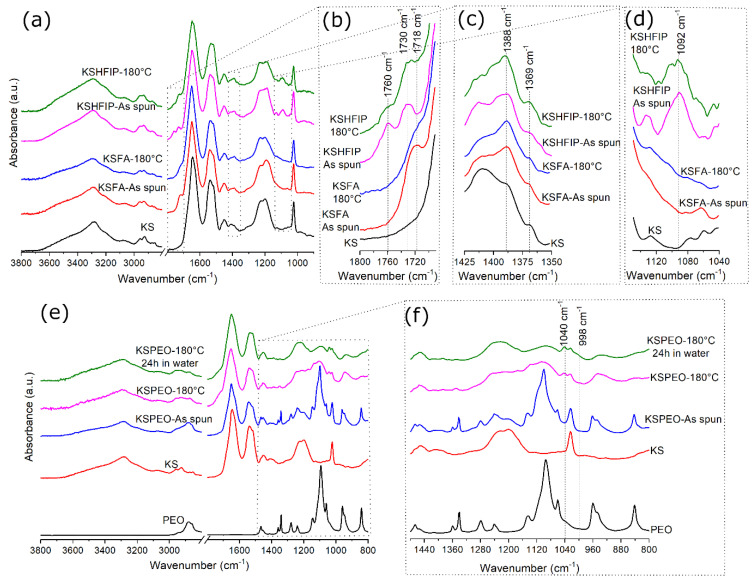
FTIR spectra before and after post-treatment at different wavenumber ranges. From (**a**–**d**): the first row corresponds to KS-NFs made of pure KS in solvents; (**e**,**f**): the second row corresponds to KS-NFs made of KS and PEO in water–KS (lyophilized powder) and PEO (powder).

FTIR spectra of all samples are reported in Figure 2. As KS spectrum is concerned, the stretching vibrations of N–H bonds at 3310 cm^−1^ is assigned to Amide A; the peak of Amide I at 1650 cm^−1^ and the peak of Amide II at 1540 cm^−1^ can be assigned to the stretching vibrations of C=O bonds and the in-plane bending modes of N–H bonds, with some contributions of C–N stretching vibrations. The peak at 1200 cm^−1^ corresponds to Amide III (an in-phase combination of N–H in-plane bending, C–N stretching vibrations, C–C stretching and C=O bending vibrations) [30]. It also relies on the nature of side-chain groups and hydrogen bonding [31]. The peak at 1025 cm^−1^ is related to the stretching vibration of Bunte salts [32].

The spectrum of PEO is characterized by bands identified as follows: CH_2_ rocking, C–C stretching, C–O stretching at 841 cm^−1^; CH_2_ twisting, CH_2_ rocking at 960 cm^−1^; CH_2_ rocking, C–C, C–O stretching at 1061 cm^−1^; C–C, C–O stretching at 1096 cm^−1^; CH_2_ rocking, C–O stretching at 1145 cm^−1^; CH_2_ twisting at 1242 and 1279 cm^−1^, CH_2_ wagging at 1341 cm^−1^; C–C stretching, CH_2_ wagging at 1358 cm^−1^ and CH_2_ scissoring at 1464 cm^−1^ [33,34,35].

It is important to remember that the solvents can destabilize the hydrogen bonds, the hydrophobic interactions and the solvation shell of proteins, as well as preferentially stabilize α–helix, β–sheet, β–turn or random coil conformation [36,37,38]. Similar variations might take place when synthetic polymers and proteins are blended. Consequently, the content of secondary structures for all NFs-membranes was estimated; the results are reported in Table 2.

**Figure 3:**
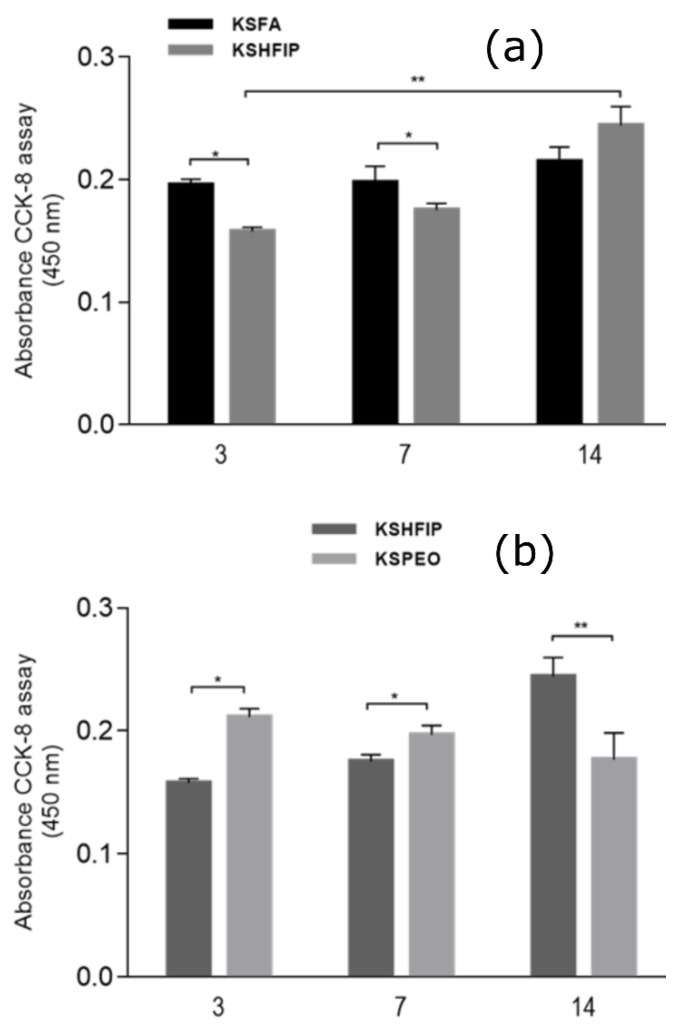
hMSCs viability cultured in KS-NFs from different solutions: (**a**) comparison of absorbance between KSFA and KSHFIP; and (**b**) KS with and without PEO. (* *p* < 0.05, ** *p* < 0.01).

The CCK-8 assay evaluated the biological response of hMSCs seeded onto KSFA, KSHFIP and KSPEO. Figure 3 shows hMSC proliferation in terms of absorbance of CCK-8 reagent. After three days, a significant increase in cell proliferation was detected in KSFA in comparison with KSHFIP. However, the latter tended to promote a more efficient hMSCs proliferation for up to 14 days compared with KSFA-NFs, whose proliferation values were almost the same over time. This result is strictly related to the contribution of HFIP and FA used as solvents. Indeed, FA presents a greater permittivity than HFIP, thus promoting the formation of thinner fibres with smaller diameters in KSFA-NFs. In accordance with previous works [39], nanosized fibre diameters (i.e., KSFA-NFs) are better recognized in vitro than sub micrometric ones (i.e., KSHFIP-NFs). Meanwhile, KSHFIP-NFs tend to form a porous network with larger pores that are able to better three-dimensionally accommodate cells, thus promoting a better response in terms of hMSC proliferation at 14 days. Similar trends were recorded in the case of KSPEO-NFs fibres in comparison with KSHFIP-NFs (Figure 3b). Until day 7, KSPEO-NFs showed higher viability of hMSC, probably due to the great affinity of PEO towards water molecules. Contrariwise, PEO seems to influence secondary structures of proteins, thus partially inhibiting the bioactive role of keratin on cell proliferation at 14 days.

## 4. Discussion

In this work, the role of morphology, i.e., fibre diameter distribution and chemical properties on in vitro hMSC response, was investigated. The most homogeneous fibres with the smallest diameter distribution (i.e., maximum frequency diameter 150 nm, range 350 nm) were obtained using FA as solvent. Flat ribbons with a size (i.e., maximum frequency diameter 700 nm, range 2.58 µm) considerably greater than the former case were produced when HFIP was used. In the literature, similar results were reported in the case of silk fibroin electrospun fibres from equivalent solvents in solution [40]. Therefore, this result could be caused by a viscosity of HFIP solution greater than that of FA solution, which hinders the jet extension during the electrospinning process [40].

The fabrication of KSPEO-NFs by electrospinning has been extensively studied in the literature [17,18]. In the KSPEO case, the polymer concentration, the polymer ratio and the electrospinning conditions allowed for producing NFs with a small number of defects and the most regular diameter of NFs (according to previous works) [17]. Additionally, KSPEO samples were thermally treated to prevent the solubilisation of protein in water. After the thermal treatment, KSPEO-NFs were immersed in water for 24 h in order to remove PEO from membranes [16,18]. The post-treatments carried out in KSPEO considerably modified the morphology and diameter distribution of NFs in comparison with untreated ones. In the literature, as-spun KSPEO-NFs have been reported to have a maximum frequency diameter of 210 nm and a range of 150 nm [17], while post-treated KSPEO have shown aggregated NFs whose diameter distribution was more irregular (maximum frequency diameter 240 nm, range 560 nm). These variations are caused by the rehydration of KSPEO-NFs, the melt (about 80 °C), and thermal degradation of PEO (which starts to degrade at 180 °C in the air with a maximum mass loss at 334 °C) [18]. In the literature, thermogravimetry analysis of PEO under isothermal condition has demonstrated that the mass loss of PEO is about 35% at 305 °C for 2 h in argon [33]. Therefore, the thermal treatment of KSPEO mostly gives rise to the PEO melting and its partial decomposition, whose degradation products can be ethylene oxide, formaldehyde, acetic aldehyde, methanol, ethanol, alkenes, non-cyclic ethers (methoxymethane, ethoxyethane, and ethoxymethane), water, CO and CO_2_ [33,34].

Compared with pure KS (lyophilized powder), new peaks were found in the spectra of as-spun samples using solvents, Figure 2b,d. Above 1700 cm^−1^, as-spun KSFA-NFs showed a peak at 1715–1722 cm^−1^ while KSHFIP-NFs had a peak at 1723–1738 cm^−1^ and another at 1760 cm^−1^. The bands above 1700 cm^−1^ are associated with the COOH modes of protonated glutamic and aspartic acid side chains [41,42]. However, it is essential to remember that the use of concentrated formic acid (<80% during 24 h) in proteins can also induce reactions of *N*- or *O*-formylations in lysine and serine/threonine residues, whose aldehyde group generates a peak above 1710 cm^−1^ [43,44].

In the KSHFIP-NFs case, one additional peak was found at 1090–1100 cm^−1^, which might be assigned to the vibration of secondary alcohols (C–O stretch at 1100 cm^−1^) or ester groups (C–O–C stretch at 1100–1030 cm^−1^) [44,45,46]. In the ATR-IR analysis, the secondary alcohol of HFIP had a peak located at 1099 cm^−1^ and, consequently, solvent traces could be the source of this signal [47]. Nevertheless, the simultaneous presence of bands at 1100 cm^−1^ and 1760 cm^−1^ gives evidence of the formation of ester groups in KSHFIP-NFs [44,46]. Considering the fact that HFIP has a range of acidity comparable to FA [48], it is possible to propose that the alcohol group of HFIP, under electrospinning conditions, might also react with KS (in particular with the carboxyl side chain groups of amino acids). Finally, it was noticed that the intensity of peaks at 1369 cm^−1^ and 1388 cm^−1^ increased when FA and HFIP were used to KS-NFs electrospin in comparison with pure KS (lyophilized powder), Figure 2c. These small bands are associated with the symmetric in-plane bending vibrations of two adjacent CH_3_ groups in amino acid side chains (valine and leucine) [42]. This result demonstrates that solvents like FA and HFIP increase the exposition of aliphatic side chains, generally buried in the hydrophobic core of proteins when peptides are brought into contact with water.

KSPEO spectra show that the electrospinning process does not vary the bands associated with PEO crystallinity (the peaks at 841 cm^−1^, 1464 cm^−1^ and the triplet peak between 1000 and 1200 cm^−1^) [34,49] in comparison with pure PEO, Figure 2e,f. However, after thermal treatment, their intensities are reduced and the presence of peaks associated with amorphous structures of PEO (the weak shoulder at 998 cm^−1^ and the peak at 1040 cm^−1^) is verified [35,49].

Table 2 shows that solvents like FA and HFIP reduce the content of every β–sheet structure and increase the content of β–turn conformations. In particular, HFIP causes a more significant reduction of β–sheet II and intermolecular β–sheet than FA. Furthermore, the same solvents exhibit opposite results in random coil and α–helix conformations. That is to say, FA increased the content of random coil and reduced the α–helix content; meanwhile, HFIP manifested the opposite effect on the same structures. It is essential to highlight that HFIP is a solvent known for its capacity to establish hydrogen bonds, raise α–helix content and break β–sheet structures [38,50,51]. In the literature, citric acid has demonstrated a similar property, as it increased the α–helix content in wool-keratin films thanks to its high capacity to generate hydrogen bonds [52]. Although FA improves the processability of KS by producing smaller and more homogenous NFs than HFIP, the latter seems to be a better solvent, as it enhances the stability of some secondary structures. In terms of thermal treatment, it has a negative effect on the α–helix structures and a positive impact on the intramolecular β–sheet and random coil structures, which could decrease the diffusion of water molecules into KS-NFs.

When PEO was used to electrospin KS in an aqueous solution, a significant reduction in intramolecular β–sheet and intermolecular β–sheet was observed. Intramolecular β–sheets are formed within individual proteins, and intermolecular β–sheets are formed among separate proteins (causing protein aggregation) [23]. This result confirms that PEO interacts with polypeptide chains. reducing the contact among proteins, which prevents the formation of these structures. Furthermore, the presence of PEO during the electrospinning process considerably increaes the content of random coil and β–turn. On the other hand, the melt of PEO induces an increase of α–helix and intramolecular β–sheet (that is to say, the contacts within individual proteins increase); the latter result indicates the partial separation of PEO and proteins. In the literature, it has been demonstrated that the presence of PEO in solutions can stabilize the α–helix conformations of proteins, explaining the rise of this structure after thermal treatment [53].

As far as the biological response of hMSCs is concerned, it is worth mentioning that the poor initial response of KSHFIP may be ascribable to the effect of fibre diameters at the sub micrometric scale, which provide topological signals in vitro that are less often recognized by cells rather than fibres at the nanoscale (i.e., KSFA). Additionally, it is suggested that KSFA and KSHFIP samples provide a major exposition of aliphatic side chains that probably reduced the wettability of KS-NFs, (see Figure 2c), limiting the contact between hMSC and KS. The importance of using protein-NFs with high hydrophilicity in cell-scaffold interaction has already been demonstrated in the literature [5].

The presence of PEO into KSPEO-NFs supports the initial cell growth, thus confirming its favourable contribution on in vitro tests. However, its mechanism of cell proliferation slowed down as a function of culture time. As reported in the literature [53], the macromolecular structures of PEO not only stabilize/destabilize some secondary structures of proteins (i.e., α–helix and intramolecular β–sheet) but may also influence the interface among water molecules and proteins, due to their steric effect.

In other terms, the presence of PEO-chains promotes a better hMSC recognition onto KS-NFs-by increasing fibre hydrophilicity at the microscopic scale, but reducing cell proliferation over time by inhibiting keratin interactions on a molecular scale into the culture. Hence, KSHFIP-NFs are the best choice in terms of cell proliferation, in agreement with evidence related to the modification of secondary structures of the protein (i.e., higher content of α–helix structure).

## 5. Conclusions

The results demonstrated that, even though solvents (HFIP and FA) and synthetic water-soluble polymers (PEO) increase the processability of proteins in electrospinning, those affect to some extent the bioactivity of biomacromolecules, like wool keratin. In the HFIP case, the hydrogen bonds that enclose polypeptide chains of KS stabilize α–helix conformations and enhance the viability of wool keratin-based NFs during their interaction with hMSC. In terms of nanostructural properties, the best NFs (the most homogeneous fibres with the narrowest diameter distribution) were obtained using FA; however, hMSC proliferation was worse than the cell response observed in HFIPKS-NFs. PEO demonstrated a dual effect on in vitro cell interactions when used to produce electrospun-NFs for scaffold materials by modifying the wettability of membranes and by reducing the accessibility of solvent molecules into keratin chains. Finally, the increase of α–helix conformations in KS-NFs seems to enhance the cell viability of hMSC over time.

## Figures and Tables

**Table 1:** Electrospinning conditions–all samples were electrospun for 1 h at 22 °C and 28% RH.

Sample	Solvent	Total Polymer Conc. (%wt.)	Polymer Blend (%wt.)	Flow Rate (mL min^−1^)	Tip-Collector Distance (cm)	Voltage (kV)	Needle i.d. (mm)
KSFA	FA	15	100 KS	0.002	15	25	0.2
KSHFIP	HFIP	6	100 KS	0.002	15	25	0.9
KSPEO ^1^	Water	7	70/30 KS/PEO	0.010	20	25	0.2

^1^ KSPEO samples were post-treated to remove PEO as reported in the literature [18].

**Table 2:** Protein secondary structure content (%).

Sample	Intermolecular β–Sheet	Intramolecular β–Sheet	β–Sheet II	β–Turn	Random Coil	α–Helix
KS	23.9	6.5	24.2	11.3	6.7	27.4
KSFA-As spun	21.0	4.7	21.1	20.5	25.0	7.7
KSFA-180 °C	20.8	6.1	17.8	20.6	27.7	7.0
KSHFIP-As spun	18.3	6.3	17.5	21.2	4.0	32.6
KSHFIP-180 °C	19.0	7.1	18.9	21.0	4.1	29.9
KSPEO-As spun	20.5	0.2	25.2	26.8	19.5	7.7
KSPEO-180 °C	16.7	13.9	21.9	17.5	16.4	13.6
KSPEO-180 °C, 24 h in water	23.0	4.9	19.0	22.9	18.2	12.0

## Data Availability

Not applicable.
